# Result of intravitreal aflibercept injection for myopic choroidal neovascularization

**DOI:** 10.1186/s12886-021-02088-x

**Published:** 2021-09-22

**Authors:** Shih-Lin Chen, Pei-Ling Tang, Tsung-Tien Wu

**Affiliations:** 1 Department of Ophthalmology, Kaohsiung Veterans General Hospital, No.386, Dazhong 1st Rd., Zuoying Dist., 813 Kaohsiung, Taiwan; 2 Section of Research Center of Medical Informatics, Kaohsiung Veterans General Hospital, Kaohsiung, Taiwan; 3 School of Medicine, National Yang Ming University, Taipei, Taiwan

**Keywords:** Aflibercept, Choroidal neovascularization, High myopia, Optical coherence tomography angiography

## Abstract

**Background:**

The current study aimed to evaluate the efficacy of intravitreal aflibercept injections as the primary treatment for subfoveal/juxtafoveal myopic choroidal neovascularization (CNV) by using optical coherence tomography (OCT). Optical coherence tomography angiography (OCTA) was further used for some patients to detect the changes of CNV after treatment.

**Methods:**

In the present study, 21 treatment-naive eyes of 21 patients with subfoveal/juxtafoveal myopic CNV received primary intravitreal aflibercept injections and were under follow-up for a minimum duration of 12 months. Among the 21 patients, 12 underwent OCTA to evaluate the changes in central foveal thickness, selected CNV area, and flow area.

**Results:**

The mean best-corrected visual acuity (BCVA) pertaining to all the patients significantly improved from the baseline value of 0.7 to 0.3 logMAR after treatment for 12 months (*P* = 0.001). However, the improvements in the median BCVA after treatment for three and 12 months were not statistically significant in the younger group (< 50 years), compared to the older group (≥ 50 years). One aflibercept injection resolved the CNV in 47.6% (10/21) of the patients. The younger group displayed greater improvement in the median central foveal thickness, compared to the older group.

OCTA revealed interlacing or disorganized pattern at the level of the outer retinal layer in 12 subjects with myopic CNV. After 3 months of treatment, both groups displayed a decrease in the size of the selected CNV area and flow area. The interlacing group displayed a trend towards better anatomical improvements.

**Conclusion:**

Intravitreal aflibercept injection provides long-term improvement in visual acuity in patients with myopic CNV. Eyes with the interlacing pattern on OCTA displayed a greater decrease in size and flow after aflibercept injection.

**Trial registration:**

Before data collection, written informed consent was obtained from each participant, whose identity information was protected by encryption and conversion to a non-identifiable format and removing data links. This study was approved by the Institutional Review Board of Kaohsiung Veterans General Hospital (KSVGH21-CT1–17).

## Background

Pathologic myopia is defined as a refractive error of − 6.0 diopters or worse spherical equivalent, accompanied by the characteristic degenerative changes in the sclera, choroid, and retinal pigment epithelium, with severe visual impairment [1, 2]. The Asian population displays a higher prevalence of pathologic myopia, ranging from 0.9 to 3.1%, compared to other regions of the world. Pathologic myopia has been reported to be the major cause of visual impairment or low vision in 12 to 27% of the Asian population [3–5].

The complications associated with pathologic myopia include posterior staphyloma, myopic maculopathy, myopic choroidal neovascularization (CNV) [1]. Myopic CNV is one of the most common complications associated with the aforementioned condition that may cause severe visual impairment [6]. It has been estimated that 5.2 to 11.3% of the patients with pathologic myopia will develop myopic CNV and the natural course and prognosis of subfoveal CNV are generally poor [3, 7–9].

Currently, therapy involving the intravitreal injection of anti-vascular endothelial growth factors (VEGFs), such as bevacizumab, ranibizumab and aflibercept, is widely used for the treatment of myopic CNV [10–17]. Recently, aflibercept, a novel recombinant fusion protein binding all isoforms of VEGF, was approved for the treatment of CNV secondary to pathologic myopia, following the well-tolerated and effective results demonstrated by the MYRROR study [18]. Traditionally, the diagnosis of myopic CNV relied on fundus fluorescein angiography, indocyanine green angiography, and optical coherence tomography (OCT). Optical coherence tomography angiography (OCTA) is a recent, noninvasive method without dye injection, which provides a layered image to observe the different shapes and sizes of CNV [19–21]. The ability of OCTA to detect the morphological features of myopic CNV have been reported by few studies [22]. A previous study used OCTA to assess the therapeutic effects of intravitreal ranibizumab injection on myopic CNV [19]. Current study is the first one to assess the one-year visual outcomes in patients with myopic CNV who underwent treatment with aflibercept and evaluations using OCTA.

## Methods

The current, retrospective study involved the patients with subfoveal/juxtafoveal CNV secondary to pathologic myopia who received intravitreal aflibercept injections. The patients underwent treatment during the time period from August 2015 to June 2020 in the Department of Ophthalmology, Kaohsiung Veteran General Hospital, Taiwan. Informed consent was obtained from all the patients. The study program was reviewed and approved by the Institutional Review Board of Kaohsiung Veterans General Hospital (KSVGH21-CT1–17).

The study included 21 patients with pathologic myopia and subfoveal/juxtafoveal CNV. The inclusion criteria included the following: treatment-naïve patients who were under follow-up for a minimum of 12 months; myopia with a spherical equivalent refractive error ≤ − 6 D or axial length ≥ 26.5 mm; active CNV secondary to pathologic myopia defined as presence of leakage from CNV seen by fluorescein angiography, and presence of intraretinal edema or subretinal fluid (SRF) or increase of central foveal thickness (CFT) seen by spectral-domain optical coherence tomography (SD-OCT); subfoveal/juxtafoveal CNV; and a best corrected visual acuity (BCVA) of 20/800 or greater. The exclusion criteria included the following: prior treatments for CNV, including PDT and thermal laser photocoagulation; history of intraocular surgery, except cataract surgery; extrafoveal CNV; CNV secondary to ocular pathology other than pathologic myopia, such as age-related macular degeneration, choroiditis, angioid streaks, or trauma; and hereditary diseases in the eye under investigation or the contralateral eye [23].

Complete ophthalmic examinations were performed at the baseline and all subsequent visits, which included Snellen BCVA (converted to the logarithm of the minimum angle of resolution; logMAR), slit-lamp biomicroscopy, tonometry, fundus examination, fluorescein angiography, and SD-OCT (RTVue XR Avanti with AngioVue, Optovue, Inc., Fremont, CA).

OCTA was conducted using RTVue XR Avanti with AngioVue at 70000 A-scans per second containing 304 × 304. The 3 × 3 mm scanning area focused on the macula and was acquired using the split-spectrum amplitude-decorrelation angiography technique. The OCTA images were automatically divided into four layers: superficial, deep, outer retina, and choroid capillaries [19]. Automatic retinal segmentation was performed using the software in the machine. Four retinal layers are classified by the software: the superficial vascular plexus (from the inner limiting membrane to the inner plexiform layer), the deep vascular plexus (between the inner and outer boundaries, respectively, at 15 and 70 mm beneath the inner plexiform layer), the outer retina (from the axonal outer plexiform layer to the Bruch membrane), and the choriocapillaris (from 31 to 59 mm inferiorly to the RPE). OCTA images were collected at the level of the outer retinal layer.

However, distortion of the retinal tissue boundaries resulted from high myopia often makes automated boundary segmentation more difficult. Manual segmentation and adjustment by the clinician were necessary at times to identify the neovascular network. The CNV area was manually contoured and measured in mm2 using the internal software of the device. Then, the flow area of the area selected manually was measured automatically in mm2, too. The values pertaining to the selected CNV areas were recorded, according to the selected size of CNV. The values pertaining to the flow area of CNV were automatically measured from the flow signals detected within the selected area. All the patients underwent OCT and OCTA prior to injection, and after one, three, and 12 months after the injection. The current study excluded the data pertaining to nine of the 21 patients, on account of poor quality or loss of original image from the database, which made further analysis impractical. Intravitreal injections of aflibercept 2 mg were administered under aseptic conditions using a 30-gauge needle, 3.5 mm or 4 mm from the limbus. Retreatment using aflibercept was performed on the basis of at least one of following observations: an increase in the central foveal thickness (CFT) of more than 50 μm between successive examinations, new or persistent cystic retinal changes, subretinal fluid, and new or persistent CNV on OCT or hemorrhage.

Continuous variables with normal distribution were described as mean and standard deviation, and continuous variables with non-normal distribution were described as median and interquartile range (IQR). An analysis of the normality of independent variables in the different groups (using the Shapiro-Wilk test) revealed that the results of the current study do not follow a normal distribution. Paired data were analyzed using the Wilcoxon signed-rank and two-way ANOVA (Friedman) tests. The significance of the differences between the values pertaining to the study groups were evaluated through further analysis. A significance level of 5% was adopted for the decision-making in statistical tests. Data were analyzed using the SPSS version 22.0 (IBM Corporation, Armonk, NY, USA).

## Results

The study involved 21 eyes pertaining to 21 patients (15 females and 6 males). The baseline clinical characteristics pertaining to the aforementioned patients are shown in Table 1. The baseline mean age pertaining to all the patients included in the present study (± standard deviation [SD]) was 51 years (± 18.5 years; range: 43 to 61.5 years). The present study detected CNV in thirteen right eyes (61.9%) and eight left eyes (38.1%). The mean axial length was 29.34 mm (± 2.72 mm; range: 28.08 to 30.80 mm). The mean duration of follow-up was 16.6 months.

**Table 1:** Baseline demographic and clinical characteristics pertaining to all the patients

Characteristics	***N =*** 21
	n (%)/Md ± IQR [range]
**Age (y)**	51.00 ± 18.50 [43.00–61.50]
**Age**	
≦ 50	10 (47.6)
> 50	11 (52.4)
**Sex**	
Male	6 (28.6)
Female	15 (71.4)
**Laterality**	
OD	13 (61.9)
OS	8 (38.1)
**AXL (mm)**	29.34 ± 2.72 [28.08–30.80]
**AXL (mm)**	
< 30	13 (61.9)
≧ 30	8 (38.1)
**CNV location (mm)**	
Subfoveal	16 (76.2)
Juxtafoveal < 200	5 (23.8)
**Number of injections**	2.00 ± 1.00 [1.00–2.00]
**Number of injections and patients**	
Once	10 (47.6)
Twice	9 (42.9)
Three times	2 (9.5)

*AXL* Axial length, *CNV* Choroidal Neovascularization, *IQR* Interquartile range, *JUXTA* Juxtafoveal, *Md* Median, *OD* Oculus Dexter, *OS* Oculus Sinister, *SUBF* Subfoveal

**Fig. 1:**
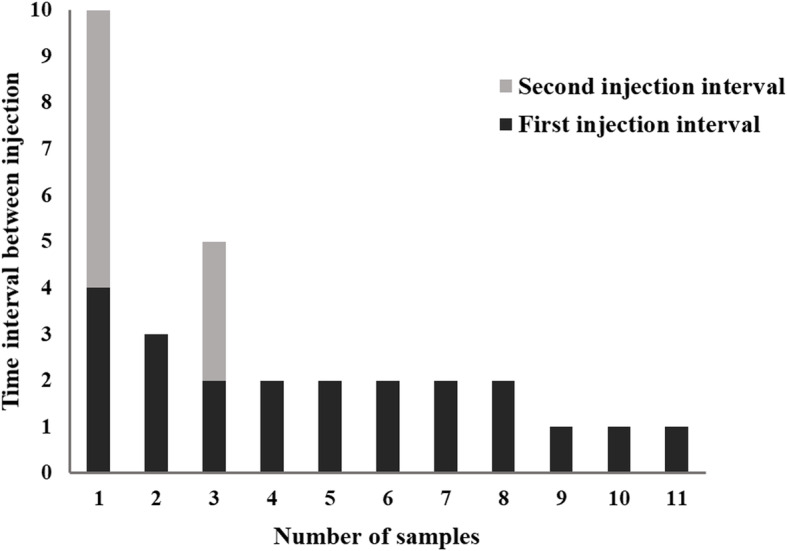
Among the eleven patients who required more than one injection, ten patients received the second injection within three months after the initial injection. Only a single patient received a third injection after a time interval of more than 3 months

The study observed that myopic CNV resolved in 10 of the 21 patients (47.6%) after one aflibercept injection. Furthermore, among the 21 patients under study, nine (42.9%) received two aflibercept injections and two (9.5%) received three aflibercept injections during the follow-up (mean of two aflibercept injections per patient). In addition, among the 11 patients who required more than one injection, 10 patients received the second injection within 3 months after the primary injection and only a single patient received a third injection 6 months after the second injection (Fig. 1).

In all the patients, the mean CFT was observed to decrease significantly from the baseline value of 285.5 μm to 249 μm after 12 months (*P* = 0.009). The mean BCVA (± standard deviation; SD) significantly improved from the baseline value of 0.7 to 0.39 logMAR after 3 months, and 0.3 logMAR after 12 months (*P* = 0.001) (Table 2). The greatest improvement in visual acuity within the first 3 months after the initial aflibercept injection, and the BCVA remained stable 12 months after the injection.

**Table 2:** Clinical characteristics pertaining to all the patients at the baseline and 12 months after the administration of the primary aflibercept injection

Parameter	Median	IQR [range]	Min-Max	***p***
**Median central foveal thickness (**μm**)**				
Baseline	285.50	41.5 [265–306.5]	222–437	0.009 ^a^
12 months after aflibercept injection	249.00	54 [223–277]	188–466	
**BCVA (logMAR)**				
Baseline	0.700	0.5 [5–1.0]	0.30–1.78	0.001 ^b^
Three months after aflibercept injection	0.39	0.3 [0.2–0.5]	0.16–0.70	
Twelve months after aflibercept injection	0.300	0.28 [0.16–0.44]	0.05–0.70	

*BCVA* best-corrected visual acuity, *IQR* interquartile range, *Max* maximum, *Min* minimum

^a^ Wilcoxon signed-rank test

^b^ Friedman two-way ANOVA test

**Fig. 2:**
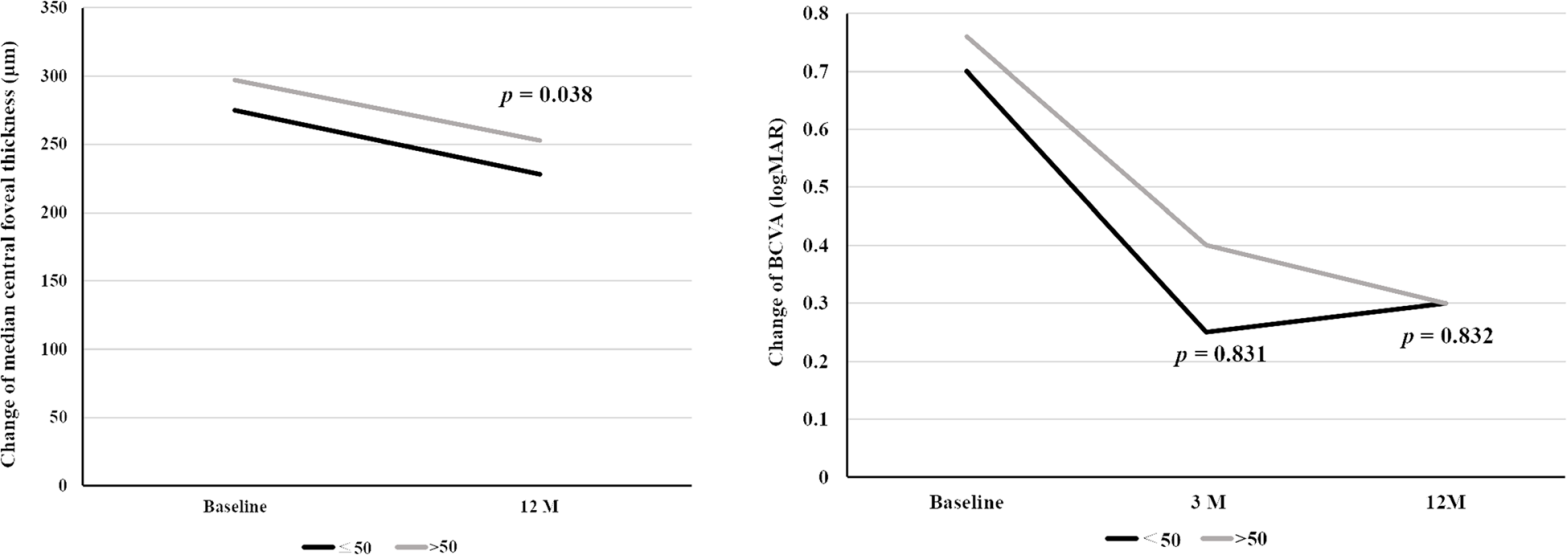
Both the younger (< 50 years) and older (≥ 50 years) groups displayed improvement in the median CFT and BCVA (logMAR). Mann-Whitney U test revealed that the improvement in median central foveal thickness was greater in the younger group, compared with the older group at the twelfth month (−52 um against − 30 um, respectively; *p* = 0.038). However, the improvement in median BCVA (logMAR) in the two groups, at the third and twelfth months, were not statistically significant (− 0.42 logMAR against − 0.34 logMAR, respectively; P = 0.0832)

Further analysis of the 21 eyes revealed that 10 eyes belonged to the patients below the age of 50 years and 11 eyes belonged to patients above or at the age of 50 years. The improvement in median CFT was greater in the younger group (< 50 years), compared to the older group (≥ 50 years) (− 52 μm against − 30 μm, respectively; *P* = 0.038) (Table 3). However, the improvement in median BCVA at the third and twelfth months were not statistically significant in the younger group, compared to the older group (− 0.42 logMAR against − 0.34 logMAR, respectively; *P* = 0.0832) (Fig. 2).

**Table 3:** Comparison between the younger group (≦50 years) and older group (> 50 years) regarding the changes in median CFT and BCVA (logMAR)

Parameter		≦ 50 years			> 50 years		***p***^**a**^
	Median	IQR [range]	Min-Max	Median	IQR [range]	Min-Max	
**Change in median CFT (μm)**	−52.00	78.75 [− 106.75- -28.00]	− 109-4.00	− 30.00	26 [− 33.00- -7.00]	−1.28- -0.10	0.038
**Change in BCVA (logMAR)**							
Three months after aflibercept injection	− 0.42	0.88 [− 0.78- -0.10]	−1.10-0.01	− 0.30	0.36 [− 0.46- -0.10]	− 1.28- -0.10	0.831
Twelve months after aflibercept injection	− 0.42	0.71 [− 0.81- -0.10]	− 1.25-0.16	− 0.34	0.64 [− 0.84- -0.20]	−73.00-45.00	0.832

^a^ Mann-Whitney U test

*BCVA* best-corrected visual acuity, *IQR* interquartile range, *Max* maximum, *Min* minimum

**Fig. 3:**
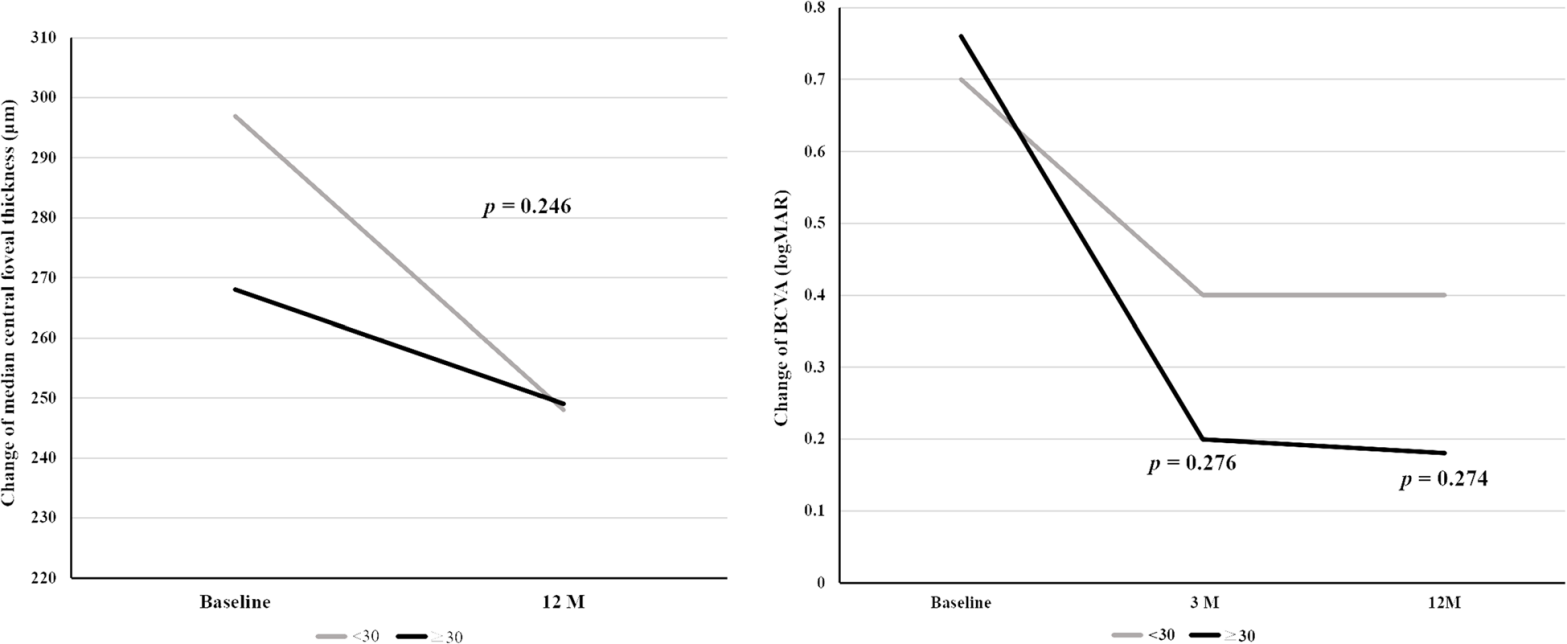
Both the groups with axial length above or below 30 mm displayed improvement in the median central foveal thickness and best-corrected visual acuity (logMAR). The Mann-Whitney U test revealed that the improvement in median central foveal thickness and the median BCVA (logMAR), in both the groups at the third and twelfth months, were not statistically significant

A comparison of the therapeutic effects of intravitreal aflibercept injection between the two groups of patients with axial length below or above 30 mm using the Mann-Whitney U test revealed that the improvement in median CFT and BCVA, in the two groups at the third and twelfth months, were not statistically significant (Table 4, Fig. 3).

**Table 4:** Comparison between the two groups with AXL < 30 mm and AXL≧30 mm regarding the changes in median CFT and BCVA (logMAR)

Parameter		AXL < 30 mm			AXL≧ 30 mm		***p***^**a**^
	Median	IQR [range]	Min-Max	Median	IQR [range]	Min-Max	
**Change in median CFT (**μm**)**	−32.00	59.00 [−64.00- -5.00]	− 109.00-45.00	−45.50	77.75 [−97.75- -20]	− 109- -7.00	0.246
**Change in BCVA (logMAR)**							
Three months after aflibercept injection	− 0.30	0.48 [− 0.58- -0.10]	−1.28-0.01	− 0.48	0.51 [− 0.74- -0.23]	− 1.10- -0.1	0.276
Twelve months after aflibercept injection	− 0.30	0.66 [− 0.81- -0.15]	− 1.28-0.16	− 0.52	0.50 [− 0.83- -0.33]	−1.25- -0.10	0.274

*BCVA* best-corrected visual acuity, *IQR* interquarti

^a^ Mann-Whitney U test

**Fig. 4:**
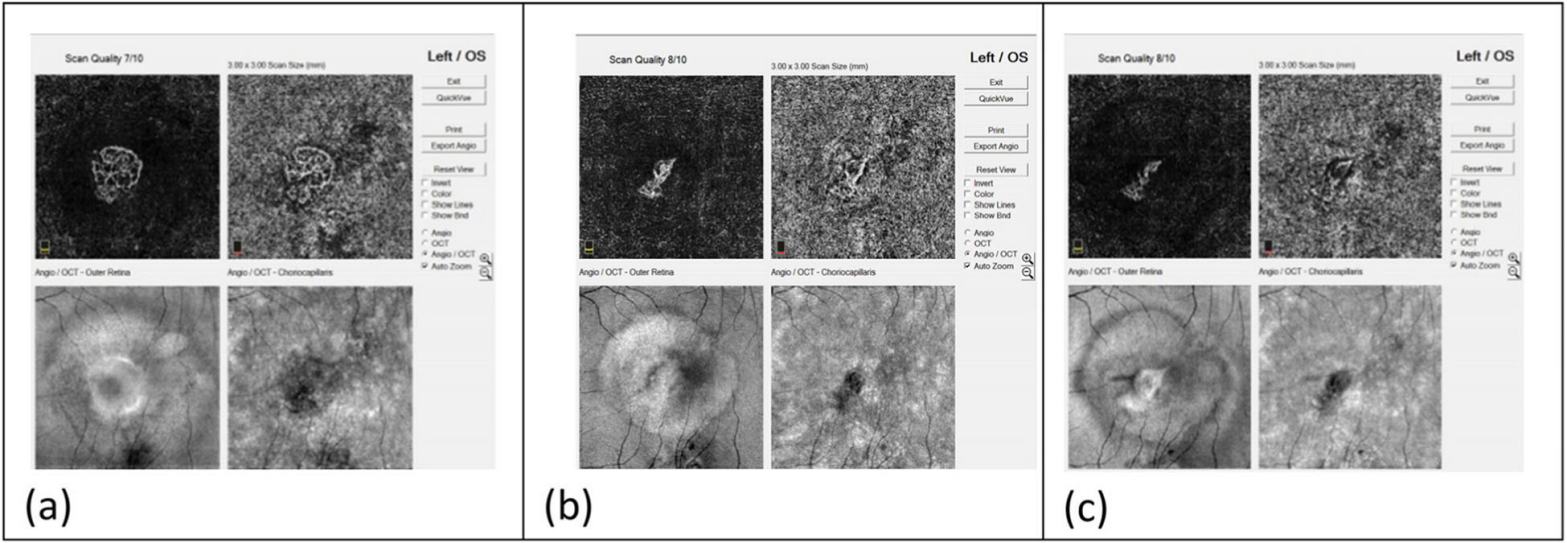
Changes in the interlacing CNV after intravitreal aflibercept injection, detected through OCTA images. **a** Dense, hyperintense vascularity with well-circumscribed, interlacing appearance was observed before treatment. **b** One month after treatment, the lesion displayed shrinkage in peripheral vascularity and reduction in the vessel density. **c** After 12 months, the CNV lesion displayed a greater decrease in the size and vessel density

**Fig. 5:**
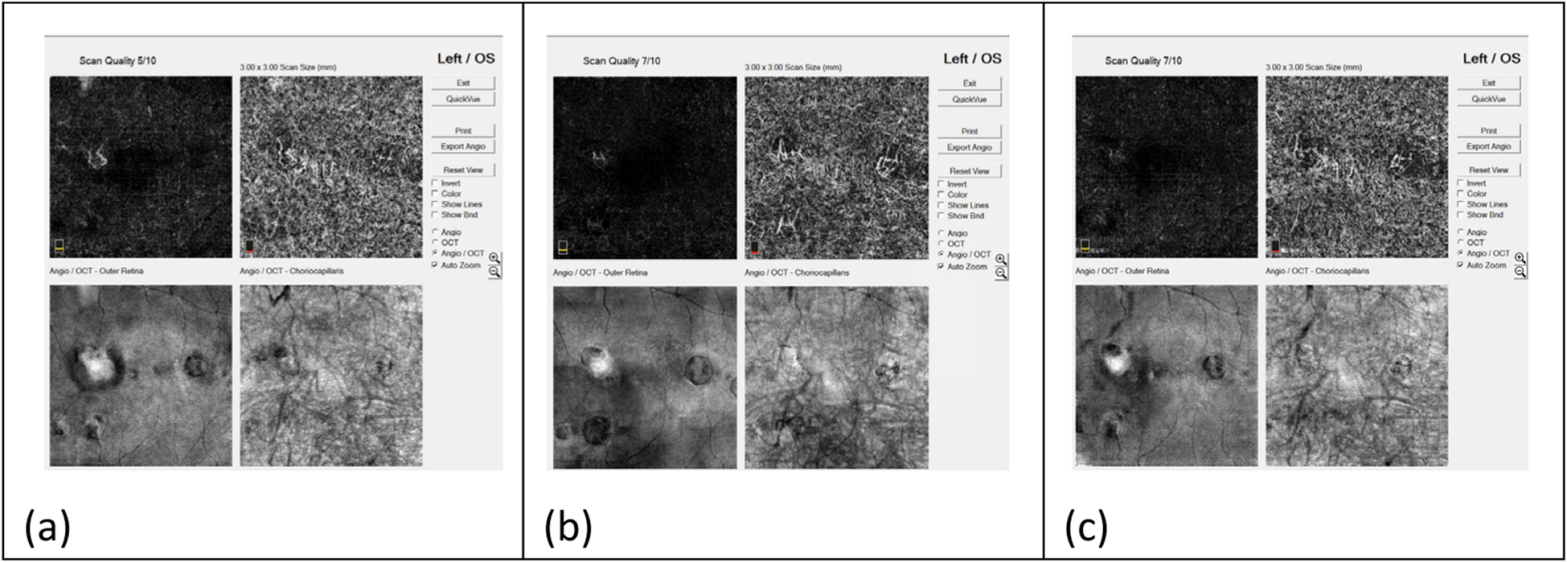
Changes in the disorganized CNV after intravitreal aflibercept injection, detected through OCTA images. **a** A small, high-flow network, harboring a vascular, loop-like pattern with no obvious capillary ends was observed before treatment. **b** One month after treatment, the lesion displayed shrinkage and reduction in vessel density. **c** After 12 months, the CNV lesion displayed a greater decrease in the size and vessel density

Myopic CNV presented as an irregular or round, closed lesion at the outer retinal layer on OCTA. This study identified two neovascular patterns: interlacing and disorganized patterns. In the interlacing pattern, which was observed in six among the twelve eyes (50%), OCTA images revealed dense vascular hyperintensity with a well-circumscribed appearance (Fig. 4). This pattern revealed a high-flow network, comprising a small capillary with a feeder vessel and small capillary ramifications. In the disorganized pattern, which was observed in six among the twelve eyes (50%), OCTA images revealed a small, high-flow network, harboring a vascular loop-like pattern with no obvious capillary ends (Fig. 5).

The OCTA images revealed changes in the group of eyes with the interlacing CNV after intravitreal aflibercept injection. One month after the treatment, shrinkage of peripheral vascularity and reduction in vessel density from previous, dense, hyperintense vascularity with well-circumscribed interlacing lesion was observed. The CNV lesion displayed a greater decrease in size and vessel density after 12 months (Fig. 4). In the group of disorganized CNV, OCTA showed shrinkage of the lesion 1 month after treatment. Reduction in vessel density from a small, high-flow network, harboring a vascular loop-like pattern with no obvious capillary ends was also observed in theses lesions. The CNV lesion showed a greater decrease in size and vessel density after 12 months (Fig. 5).

**Fig. 6:**
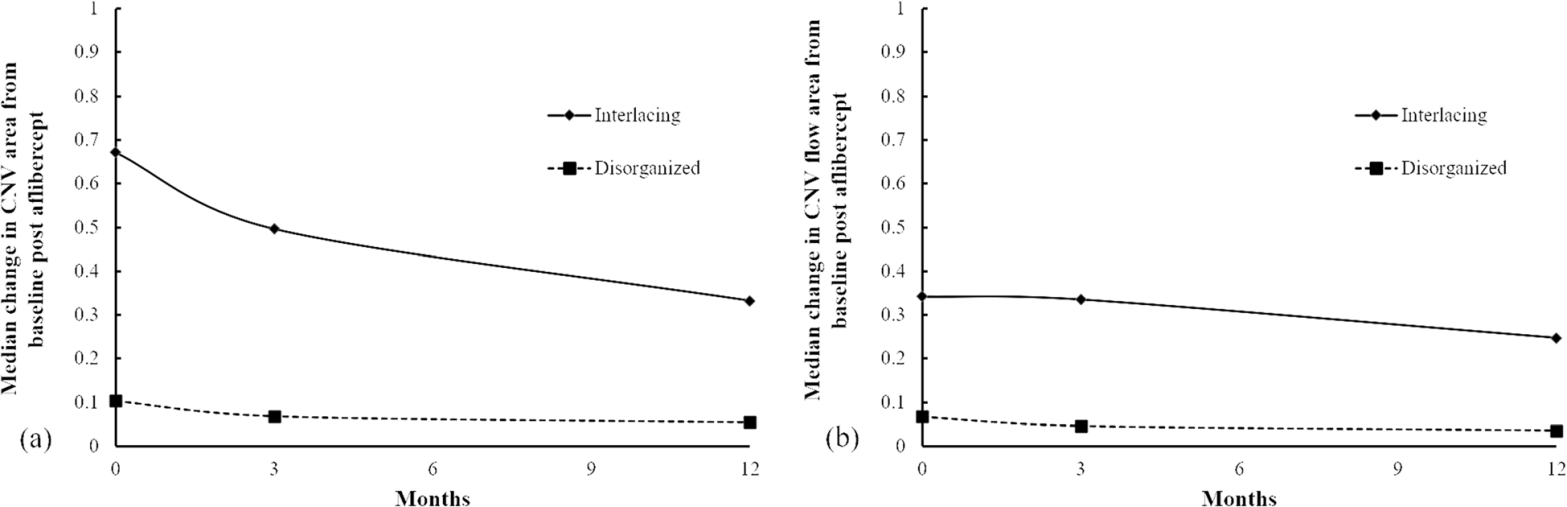
**a, b** Three months after the aflibercept injection, a decrease in both the CNV area and flow area were observed in both interlacing and disorganized patern and the treatment effects persisted until the twelfth month. Figure 6 **a**. Much more decrease in the CNV area and flow area, at the third and twelfth months, were observed in interlacing pattern

Three and twelve months after the primary injection, the size of the selected CNV area and the flow area revealed a trend towards decline in both the interlacing pattern and the disorganized pattern (Table 5) (Fig. 6(a)(b)). The interlacing pattern displayed a trend towards better improvement in the size and flow of the selected CNV area (Fig. 6(a)(b)). However, the present study did not observe any obvious difference between the two groups with different patterns with regard to the improvement in BCVA after intravitreal aflibercept injection (Table 5).

**Table 5:** Demographic characteristics and changes in the features pertaining to OCTA images after aflibercept treatment in patients with myopic CNV

CNV Pattern	No.	Age	Injection time	CFT (mm)		BCVA			CNV area (mm^**2**^)			CNV flow area (mm^**2**^)		
Time ^**a**^				Pre	12 M (△)	Pre	3 M (△)	12 M (△)	Pre	3 M (△)	12 M(△)	Pre	3 M (△)	12 M (△)
Interlacing	1	48	1	302	193 (− 109)	1.48	0.7 (− 0.78)	0.7 (− 0.78)	0.701	0.656 (− 0.05)	0.406 (− 0.29)	0.296	0.407 (0.11)	0.267 (− 0.03)
Interlacing	2	58	1	271	238 (− 33)	0.7	0.4 (− 0.30)	0.4 (− 0.30)	0.402	0.336 (− 0.07)	0.259 (− 0.14)	0.266	0.265 (0.00)	0.228 (− 0.04)
Interlacing	3	20	2	305	211 (−94)	0.3	0.31 (0.01)	0.46 (0.16)	0.96	0.854 (−0.11)	0.739 (− 0.22)	0.782	0.658 (− 0.12)	0.610 (− 0.17)
Interlacing	4	24	1	355	249 (−106)	1.3	0.2 (−1.10)	0.05 (− 1.25)	0.641	0.245 (−0.40)	0.168 (−0.47)	0.389	0.13 (−0.26)	0.146 (− 0.24)
Interlacing	5	29	2	286	230 (−56)	0.5	0.16 (−0.34)	0.16 (−0.34)	0.484	0.202 (−0.28)	0.172 (− 0.31)	0.296	0.148 (− 0.15)	0.160 (− 0.14)
Interlacing	6	46	1	261	188 (−73)	0.5	0.2 (−0.30)	0.1 (−0.40)	0.846	0.668 (−0.18)	0.656 (− 0.19)	0.674	0.459 (− 0.22)	0.459 (− 0.22)
Disorganized	7	46	3	302	193 (−109)	1.48	0.7 (−0.78)	0.7 (−0.78)	0.061	0.034 (−0.03)	0.018 (− 0.04)	0.026	0.018 (− 0.01)	0.008 (− 0.02)
Disorganized	8	48	1	282	250 (−32)	0.7	0.16 (−0.54)	0.16 (−0.54)	0.119	0.088 (−0.03)	0.075 (− 0.04)	0.067	0.046 (− 0.02)	0.038 (− 0.03)
Disorganized	9	51	1	222	226 (4)	0.5	0.4 (−0.10)	0.4 (−0.10)	0.166	0.077 (−0.09)	0.054 (− 0.11)	0.131	0.054 (− 0.08)	0.047 (− 0.08)
Disorganized	10	62	2	362	407 (45)	1.78	0.5 (−1.28)	0.5 (−1.28)	0.161	0.097 (−0.06)	0.067 (−0.09)	0.136	0.071 (−0.07)	0.039 (− 0.10)
Disorganized	11	63	2	297	267 (−30)	0.4	0.3 (−0.10)	0.2 (−0.20)	0.077	0.06 (−0.02)	0.032 (− 0.05)	0.022	0.021 (− 0.00)	0.02 (− 0.002)
Disorganized	12	76	3	281	248 (−33)	1	0.16 (−0.84)	0.16 (−0.84)	0.088	0.053 (−0.04)	0.055 (− 0.03)	0.037	0.043 (0.01)	0.033 (−0.004)

*BCVA* best-corrected visual acuity, *CFT* central foveal thickness, *CNV* choroidal neovascularization, *logMAR* logarithm of the minimum angle of resolution, *M* month, *Pre* Pre-treatment, *(△)* Changes compared with baseline before aflibercept treatment

^a^ 3 M: three months after aflibercept treatment; 12 M: twelve months after aflibercept treatment

## Discussion

In the current study, most eyes showed improvement in vision and the burden of intravitreal therapies was found similar to the MYRROR study, which revealed that the patients with myopic CNV who underwent intravitreal aflibercept injection displayed significant improvements in visual and anatomic parameters over a time period of 48 weeks [18].

After intravitreal aflibercept injections, the subjects in the younger group displayed a greater reduction in CFT at 12 months compared to the order group. The aforementioned observation was concordant with the clinical findings reported by Yoshida et al. and Bruè et al. [7, 24] At 3 and 12 months, the younger group displayed a trend towards better improvement in vision after intravitreal aflibercept injections, although the results were not statistically significant.

A less reduction in CFT at 12 months after treatment associated with myopic CNV in the older age group could be attributed to the decreased integrity and function of the myopic retinal pigment epithelium that downgrades the inhibition of angiogenesis [25]. Indeed, some studies report that myopic CNV in older patients could manifest simultaneous AMD and high myopia, resulting in poor natural outcomes. Moreover, older patients tend to develop chorioretinal atrophy or degeneration that negatively influences the final visual acuity. However in our study, no statistically significant difference in BCVA changes at 12 months after treatment between younger and older group is noted.

Previous studies reported that the role of spherical equivalent refraction and axial length as predictive factors associated with the final visual acuity after anti-VEGF treatment in myopic CNV remained controversial. Bruè et al. reported that higher myopia is associated with decreased visual acuity after intravitreal aflibercept injections in young patients with myopic CNV. Some studies identified a significant positive correlation between BCVA and macular choroidal thickness after anti-VEGF treatment [23]. Greater axial length was often considered to be major factor associated with macular choroidal thinning. The current study did not observe any difference in the treatment outcomes between the groups with long and short axial length (Table 4). This could be attributed to the relatively small sample size and short duration of follow-up. Further investigations should be performed to elucidate the significance of axial length in predicting treatment outcomes.

Recently, OCTA is being widely used in the diagnosis of various macular diseases and provides detailed, layered images of enface retinal and choroidal vasculature [26–29]. Cheng et al. used OCT B-scan and OCTA to perform quantitative analysis and monitor the therapeutic effects of intravitreal ranibizumab injection (0.5 mg/0.05 mL) (Lucentis; Genentech, Inc., South San Francisco, CA) in myopic CNV [19]. The OCTA revealed significant attenuation of the capillaries and small caliber feeder vessels after intravitreal ranibizumab injection. Moreover, the study reported a simultaneous reduction in the size of the selected CNV area and flow area.

To the best of our knowledge, this is the first study that used OCTA to detect the changes in the shape, selected area, and flow area of CNV after intravitreal aflibercept injection at 3 and 12 months. In the current study, among twelve patients who underwent OCTA, eleven patients displayed improvement in BCVA and twelve patients displayed decrease in the selected CNV area at 3 months. The treatment effects persisted until the twelfth month. Nine in twelve patients displayed decrease in the selected CNV flow area at 3 months after aflibercept injection. While eleven in twelve patients displayed decrease in the selected CNV flow area at 12 months after aflibercept injection (Table 5). The decrease of selected CNV area and flow area after aflibercept injection is consistent with the results reported by Cheng et al., wherein myopic CNV was treated using intravitreal ranibizumab injection [19].

According to a previous study, it was often considered that active CNV presented as retinal hemorrhage, intra-retinal fluid, sub-retinal fluid, or pigment epithelial detachment, as shown by the fundus photography and OCT B-scan images. These active CNV lesions were also considered to be more sensitive to anti-VEGF therapy. However, some cases of myopic CNV that presented with scars or fibrosis on fundoscopic examinations after anti-VEGF treatment could also show residual, high reflective neovascularization lesions on OCT B-scan images. The aforementioned condition often confused clinicians regarding the necessity for further treatment and the presence of CNV activity. Hence, OCTA is expected to provide additional biomarkers and parameters to guide further management.

Bruyère et al. used OCTA to identify two types of neovascular membranes in high myopia. The first involved small, disorganized vascular loops, suggesting an immature neovascular network. The second is a larger, highly structured, interlacing network, suggesting a mature lesion [21]. Querques et al. reported that on OCTA images, active myopic CNVs could be mainly interlacing, whereas the abnormal vascular network with tangled pattern could be inactive myopic CNV [30]. Cheng et al. reported that sea-fan and lacy-wheel types tend to be clinically active CNVs, whereas poorly defined lesions, such as filamentous CNV vessels, could be either active or inactive.

In the present study, the interlacing group displayed a trend towards better improvement in CFT reduction and decrease in the selected CNV area and flow area (Table 5) (Fig. 6(a)(b)). Additionally, previous studies have reported consistent results and stated that the capillaries and small caliber feeder vessels were significantly attenuated after intravitreal ranibizumab injection, whereas the main central trunk vessel and large caliber feeder vessels remained unchanged [22, 31].

However, owing to the limited sample size, the scenario warrants further investigation to clarify the significance of CNV patterns in predicting the response to anti-VEGF therapy. OCTA could be considered as a useful tool that can be employed to identify the different CNV patterns and detect CNV activity, predict treatment response, and monitor the need for repeated treatments in patients with myopic CNV.

The current study has certain limitations. The series was relatively small, and the duration of follow-up was short. The present study observed some poor image quality with significant motion artifacts, due to unstable fixation, which could make the analysis more challenging. Similarly, the accuracy of the layers automatically divided by OCTA could be affected by the long axial length of the pathologic myopia eyeballs.

## Conclusion

In conclusion, the administration of a single injection of intravitreal aflibercept 2.0 mg at the baseline in patients with myopic CNV showed effective results. A single aflibercept injection was observed to resolve myopic CNV in approximately half of the patients. The median number of injections was two injections within 12 months.

Eyes with the interlacing pattern on OCTA displayed a greater decrease in size and flow after intravitreal aflibercept.

Further studies with large sample sizes and longer follow-ups are necessary to explore the relationship between different CNV patterns, detected through OCTA, and the therapeutic effects of anti-VEGF on myopic CNV.

## Data Availability

The datasets used and/or analysed during the current study are available from the corresponding author on reasonable request.

## Abbreviations

CNV: Choroidal neovascularization
PDT: Photodynamic therapy
VEGFs: Vascular endothelial growth factors
OCT: Optical coherence tomography
OCTA: Optical coherence tomography angiography
SD-OCT: Spectral-domain optical coherence tomography
BCVA: Best-corrected visual acuity
logMAR: Logarithm of the minimum angle of resolution
CFT: Central foveal thickness
IQR: Interquartile range
SD: Standard deviation
